# Prognostic implication of molecular subtypes and response to neoadjuvant chemotherapy in 760 gastric carcinomas: role of Epstein–Barr virus infection and high‐ and low‐microsatellite instability

**DOI:** 10.1002/cjp2.137

**Published:** 2019-06-17

**Authors:** Meike Kohlruss, Bianca Grosser, Marie Krenauer, Julia Slotta‐Huspenina, Moritz Jesinghaus, Susanne Blank, Alexander Novotny, Magdalena Reiche, Thomas Schmidt, Liridona Ismani, Alexander Hapfelmeier, Daniel Mathias, Petra Meyer, Matthias M Gaida, Lukas Bauer, Katja Ott, Wilko Weichert, Gisela Keller

**Affiliations:** ^1^ Institute of Pathology Technical University of Munich Munich Germany; ^2^ Department of Surgery University of Heidelberg Heidelberg Germany; ^3^ Department of Surgery Technical University of Munich Munich Germany; ^4^ Institute of Medical Informatics, Statistics and Epidemiology Technical University of Munich Munich Germany; ^5^ Institute of Pathology University of Heidelberg Heidelberg Germany; ^6^ Department of Surgery Klinikum Rosenheim Rosenheim Germany; ^7^ German Cancer Consortium (DKTK), Partner Site Munich Institute of Pathology Munich Germany

**Keywords:** microsatellite instability, Epstein–Barr virus, adenocarcinoma, gastric, gastro‐oesophageal junction, outcome, neoadjuvant chemotherapy, prognosis, molecular subtype

## Abstract

Epstein–Barr virus positivity (EBV(+)) and high‐microsatellite instability (MSI‐H) have been identified as molecular subgroups in gastric carcinoma. The aim of our study was to determine the prognostic and predictive relevance of these subgroups in the context of platinum/5‐fluorouracil (5‐FU) based preoperative chemotherapy (CTx). Additionally, we investigated the clinical relevance of the low‐MSI (MSI‐L) phenotype. We analysed 760 adenocarcinomas of the stomach or the gastro‐oesophageal junction encompassing 143 biopsies before CTx and 617 resected tumours (291 without and 326 after CTx). EBV was determined by PCR and *in situ* hybridisation for selected cases. MSI was analysed by PCR using five microsatellite markers and classified as MSI‐H and MSI‐L. Frequencies of EBV(+), MSI‐H and MSI‐L in the biopsies before CTx were 4.2, 10.5 and 4.9% respectively. EBV(+) or MSI‐H did not correlate with response, but MSI‐L was associated with better response (*p* = 0.011). In the resected tumours, frequencies of EBV(+), MSI‐H and MSI‐L were 3.9, 9.6 and 4.5% respectively. Overall survival (OS) was significantly different in the non‐CTx group (*p* = 0.014). Patients with EBV(+) tumours showed the best OS, followed by MSI‐H. MSI‐L was significantly associated with worse OS (hazard ratio [HR], 2.21; 95% confidence interval [CI], 1.21–4.04, *p* = 0.01). In the resected tumours after CTx, MSI‐H was also associated with increased OS (HR, 0.54; 95% CI, 0.26–1.09, *p* = 0.085). In multivariable analysis, molecular classification was an independent prognostic factor in the completely resected (R0) non‐CTx group (*p* = 0.035). In conclusion, MSI‐H and EBV(+) are not predictive of response to neoadjuvant platinum/5‐FU based CTx, but they are indicative of a good prognosis. In particular, MSI‐H indicates a favourable prognosis irrespective of treatment with CTx. MSI‐L predicts good response to CTx and its negative prognostic effect for patients treated with surgery alone suggests that MSI‐L might help to identify patients with potentially high‐benefit from preoperative CTx.

## Introduction

Pre‐/peri‐operative chemotherapy (CTx) containing a platinum/5‐fluorouracil (5‐FU) combination is recommended for patients with advanced gastric carcinoma (GC) in western countries, but response rates are limited 1, 2, 3.

Recent studies suggest that molecular classification should be considered for optimal therapy planning. Molecular classification systems have been described by The Cancer Genome Atlas (TCGA) network and the Asian Cancer Research Group (ACRG). Both include tumours with microsatellite instability (MSI) as one subgroup 4, 5. MSI is characterised by accumulation of length alterations of microsatellite sequences and is commonly determined using five microsatellite markers 6. Depending on the number of unstable markers, MSI can be classified into high‐MSI (MSI‐H) (≥2/5 unstable markers) or low‐MSI (MSI‐L) (1/5 unstable marker). If there is no MSI, the tumour is considered as microsatellite stable (MSS). MSI‐H is related to DNA mismatch repair deficiency and is detected in about 7–24% of GC 4, 5, 7, 8, 9, 10, 11. MSI‐H has been related to good prognosis in GC in the majority of studies, but conflicting results of the prognostic significance for patients treated with CTx have been described 8, 9, 11, 12, 13, 14. Specifically a negative prognostic effect of MSI‐H for patients receiving neoadjuvant CTx has been reported based on the analysis of tumours resected after CTx, whereas another study reported the MSI‐H phenotype as a favourable prognostic marker also in this therapeutic setting 9, 14.

The MSI‐L phenotype has been described in various tumours, including GC, but the biological and clinical significance is largely unclear and MSI‐L and MSS tumours are frequently combined in one group 8, 11.

Another subgroup of the TCGA classification is formed by Epstein–Barr virus positive (EBV(+)) tumours, which represent about 4–10% of GC 4, 10, 15, 16.

EBV positivity was shown to be associated with better prognosis in GC patients though there are studies that find no clear correlation between EBV status and survival and the relevance of EBV positivity to predict response to neoadjuvant CTx is unclear 10, 15.

EBV positivity and MSI‐H were associated with good response in a clinical trial evaluating an immune check point inhibitor in metastatic GC 17.

In light of these new therapeutic options and against the background of still limited data available in the literature related to neoadjuvant CTx, further knowledge about the clinical relevance of MSI and EBV status in connection with classical treatment regimens is essential for the selection of the most appropriate treatment for each GC patient.

Thus the goal of our study was to determine the prognostic and predictive significance of EBV positivity and MSI for carcinomas of the stomach and gastro‐oesophageal junction in the context of preoperative platinum/5‐FU based CTx. As various clinical aspects are relevant in this therapeutic setting, we performed a comprehensive analysis of overall 760 tumours encompassing three different patient cohorts each with specific characteristics.

First, we analysed tumour biopsies before neoadjuvant CTx; this represents the most appropriate cohort to test for an association with therapy response as it allows inclusion of both non‐responding and completely or nearly completely responding patients. In addition, biopsies are the type of specimens that are available before the start of CTx in daily clinical practice. Second, we analysed the molecular subgroups in a relatively large cohort of resected tumours from patients treated with surgery alone and third, resected tumours from patients after neoadjuvant CTx, to determine if their prognostic role is comparable and might support the choice of subsequent treatment modalities. Finally, as the clinical significance of the MSI‐L phenotype in GC is poorly characterised, we aimed to fill this gap and analysed MSI in terms of MSI‐H and MSI‐L.

## Material and methods

### Patients

**Figure 1:**
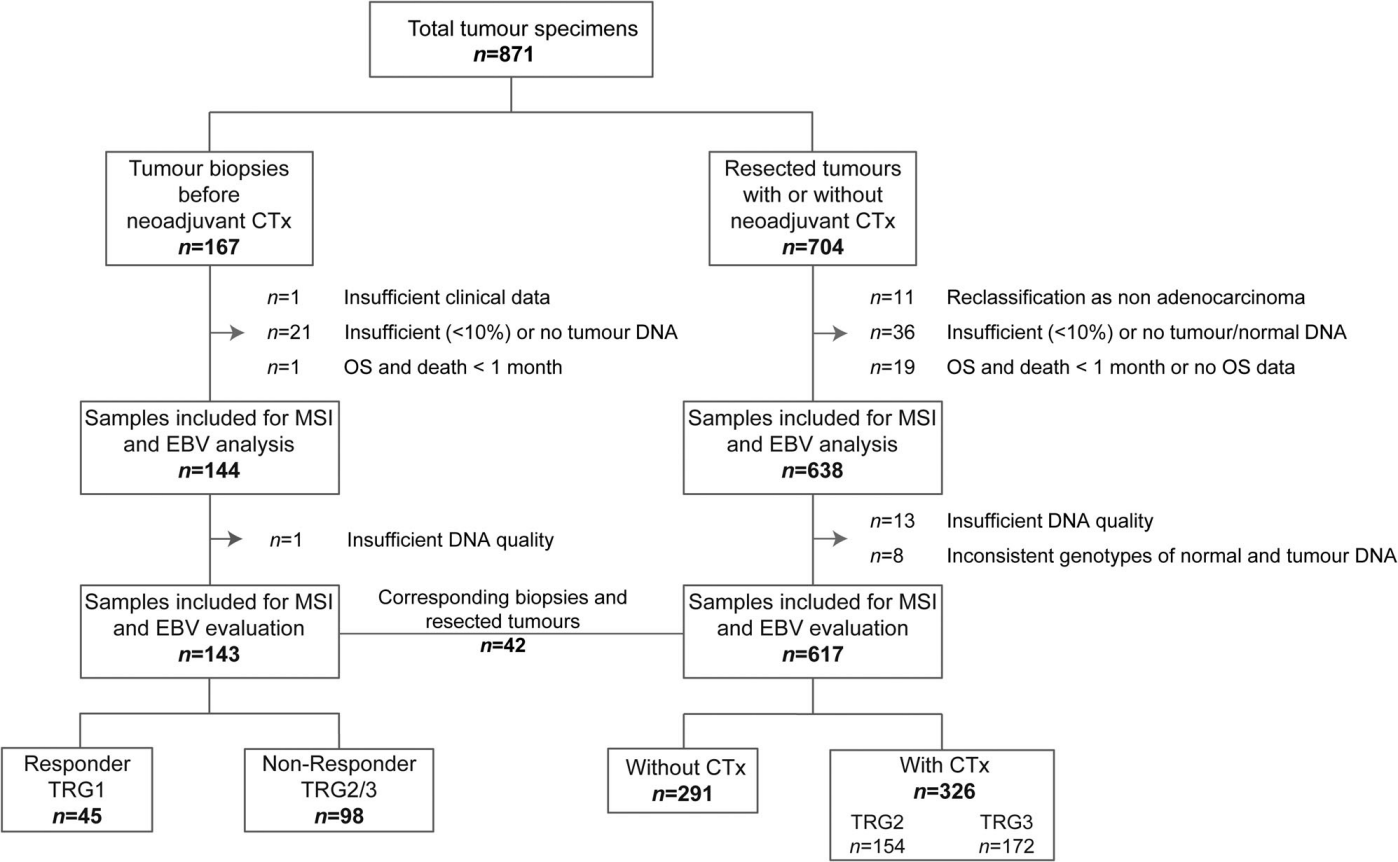
Flow chart diagram of patient and specimen inclusion.

Resected tumours from 704 patients with gastric adenocarcinomas including tumours of the gastro‐oesophageal junction (AEG II and AEG III according to Siewert and Stein 18) that were treated between 2001 and 2013 at the Department of Surgery of the University of Heidelberg and between 2001 and 2012 at the Technical University of Munich were included in the study. Essentially the patient cohort was described previously 19. Tumours from 87 patients were excluded from this study and the final cohort of 617 tumours consisted of 291 tumours from patients treated with surgery alone and 326 tumours from patients after neoadjuvant CTx (Figure 1).

Tumour biopsies before neoadjuvant CTx from 167 patients treated between 1993 and 2013 at the Department of Surgery of the Technical University of Munich were included. Limitation for inclusion was the availability of DNA or paraffin blocks with tumour and non‐tumorous tissues and 143 biopsies were finally analysed. Corresponding biopsies before and resected tumours after CTx from 42 patients were included (Figure 1). Previous studies had analysed biopsy specimens of 58 patients for MSI using a different panel of microsatellite markers 7, 20.

### CTx and surgery

Patients were treated with platinum/5‐FU based chemotherapeutic regimens as detailed in supplementary material, Table S1. Comparison of overall survival (OS) of patients with resected tumours after CTx relating to treatment with platinum/5‐FU based regimens with and without taxanes or relating to regimens containing two or three drugs, revealed no statistically significant differences in either case (see supplementary material, Table S2).

All surgical approaches included an abdominal D2 lymphadenectomy and are described in detail in supplementary material, Supplementary materials and methods.

### Response evaluation

Response to preoperative CTx was determined histopathologically and was classified into three tumour regression grades (TRG): TRG1, TRG2 and TRG3, which corresponded to <10, 10–50 and >50% residual tumour cells/tumour bed respectively. The prognostic relevance of this classification system has been demonstrated in previous studies 21, 22.

All three TRGs were present among the patients with tumour biopsies before CTx; patients with TRG1 were classified as responders, and those with TRG2 and TRG3 as non‐responders. Only tumours with TRG2 and TRG3 were present among the patients in the resected tumour cohort after CTx; these allow isolation of sufficient DNA from residual tumour cells.

### Follow‐up and overall survival

Follow‐up was performed as described 19. OS was defined as the time between the date of operation and death by any cause.

### Ethics statement

The study was in accordance with the Declaration of Helsinki and was approved by the local Institutional Review Boards at the Technical University of Munich (reference: 502/15s) and at the University of Heidelberg (reference: 301/2001).

### DNA isolation

DNA from formalin fixed paraffin embedded (FFPE) tissues was isolated after manual microdissection from 8 μm thick sections after deparaffinisation and proteinase K digestions using the Maxwell extraction system according to the instructions of the manufacturer (Promega, Madison, WI, USA). Details are described in supplementary material, Supplementary materials and methods. Only samples with a tumour cell content of at least 10% were included for MSI analysis according to the described detection limit for MSI of 2–10% tumour alleles 23.

### Analysis for MSI

MSI was determined by PCR analysing two mononucleotide repeats BAT25, BAT26 and three dinucleotide repeats D2S123, D5S346, D17S250 as recommended by the National Cancer Institute 6. Details are described in supplementary material, Supplementary materials and methods. Tumours with additional alleles at specific microsatellite markers compared to the corresponding normal tissue were classified as MSI. According to a standardised definition, MSI‐H was defined if at least two of the five markers showed MSI and as MSI‐L if one of the five markers showed MSI 6. Tumours without MSI were classified as MSS. MSI‐L cases were confirmed by a second independent PCR.

### Detection of EBV

Screening for EBV was performed by a PCR based assay using primers for amplification of EBV specific DNA in the BamHI‐W and BamHI‐K regions of the virus as described 24. Tumours with positive signals in the PCR assay were further analysed by chromogenic *in situ* hybridisation using the EBV early RNA Probe and the iViEW Blue detection kit (Ventana, Roche, Tucson, AZ, USA) on an automated system (Ventana Medical System, Roche) according to the instructions of the manufacturer. EBV positivity was defined when positive staining after *in situ* hybridisation was present in the nuclei of the tumour cells.

### Statistical analysis

Chi‐squared tests or Fisher's exact tests were used for hypothesis testing of differences between the relative frequencies. Kaplan–Meier estimates of survival rates were compared by log rank tests. Relative risks were estimated by hazard ratios (HRs) from univariable Cox proportional hazard models or from Firth's corrected Cox‐regression. A multivariable Cox proportional hazards model was built by stepwise forward variable selection using likelihood‐ratio tests of pre‐therapeutically and post‐therapeutically available clinical factors.

The pre‐therapeutically available factors were: sex, age (continuous variable), histological type according to Laurén (intestinal versus non‐intestinal), tumour localisation (proximal, middle, distal, total) and clinically determined tumour stage (cT2 versus cT3/cT4). The post‐therapeutic factors were: sex, age, histological type according to Laurén, tumour localisation, depth of tumour invasion (pT2 versus pT3/pT4), lymph node involvement (pN0 versus pN+), R‐category (R0 versus R+) and status of metastasis (M0 versus M+). Statistical analyses were performed using SPSS, Version 25 (IBM Corp., Armonk, NY, USA). Exploratory 5% significance levels (two‐tailed) were used for hypothesis testing.

## Results

### Study enrolment and patient characteristics

Our study population consisted of different GC cohorts. The biopsy cohort encompassed patients with pre‐therapeutic tumour biopsies before CTx with inclusion of responding (TRG1) and non‐responding (TRG2/3) patients to accurately determine the predictive and prognostic value of the molecular subgroups for CTx treatment. Of 167 pre‐therapeutic biopsies, which were initially evaluated for the study, 24 were excluded. Among the 143 analysed biopsies, 45 of the patients showed TRG1, 34 showed TRG2 and 64 showed TRG3 in the resected specimens. The OS of patients in relation to TRG was significantly different (log rank *p* < 0.01) and is shown in supplementary material, Figure S1. Only some difference in OS was observed between patients with TRG2 and TRG3, therefore both groups were classified as non‐responders and patients with TRG1 as responders.

The resected tumour cohort encompassed initially 704 patients and 87 were excluded. Of the remaining 617 resected tumours, 291 were from patients treated with surgery alone and 326 were from patients after treatment with CTx among them 154 with TRG2 and 172 with TRG3. An overview of the enrolment of patients with the respective exclusion criteria is shown in Figure 1. Clinical characteristics of the patients included for analysis are summarised in Table 1.

**Table 1:** Patient characteristics

				Resected specimens					
		Tumour biopsies before neoadjuvant CTx		All		Without neoadjuvant CTx		After neoadjuvant CTx	
Category	Value	*n*	%	*n*	%	*n*	%	*n*	%
Cases	Total	143	100	617	100	291	100	326	100
Age (years)	Median	61.1		64.6		68.1		61.3	
	Range	23.1–78.0		28.3–90.9		32.1–90.9		28.3–81.2	
Follow‐up period (month)	Median	69.6		57.9		58.8		56.7	
	95% CI	61.6–77.6		53.1–62.7		50.7–66.9		47.4–66.0	
Overall survival (month)	Median	48.1*		44.6		85.0		32.4	
	95% CI	26.2–70.0		30.2–59.0		51.7–118.3		23.0–41.8	
Sex	Male	109	76.2	453	73.4	193	66.3	260	79.8
	Female	34	23.8	164	26.6	98	33.7	66	20.2
Localisation	Proximal	100	69.9	301	48.8	97	33.3	204	62.6
	Middle	23	16.1	153	24.8	84	28.9	69	21.2
	Distal	14	9.8	131	21.2	92	31.6	39	12.0
	Total/linitis	6	4.2	28	4.5	14	4.8	14	4.3
	N/A	0	0	4	<1	4	1.4	0	0
Laurén histological subtype	Intestinal	72	50.3	347	56.2	155	53.3	192	58.9
	Non‐intestinal	71	49.7	270	43.8	136	46.7	134	41.1
Tumour grade	G1/2	33	23.1	125	20.3	80	27.5	45	13.8
	G3/4	110	76.9	400	64.8	210	72.5	190	58.3
	N/A	0	0	92	14.9	1	0	91	27.9
cT	cT2	8	5.6	144	23.3	129	44.3	15	4.6
	cT3/cT4	131	91.6	471	76.3	161	55.3	310	95.1
	N/A	4	2.8	2	<1	1	<1	1	<1
(y) pT†	(y) pT0	9	6.3	0	0	0	0	0	0
	(y) pT1	12	8.4	56	9.1	42	14.4	14	4.3
	(y) pT2	20	14.0	79	12.8	47	16.2	32	9.8
	(y) pT3	81	56.6	328	53.2	139	47.8	189	58.0
	(y) pT4	19	13.3	154	25.0	63	21.6	91	27.9
	N/A	2	1.4	0	0	0	0	0	0
(y) pN	Negative	61	42.7	189	30.6	104	35.7	85	26.1
	Positive	80	55.9	428	69.4	187	64.3	241	73.9
	N/A	2	1.4	0	0	0	0	0	0
Metastasis status	No	97	67.8	534	86.5	272	93.5	262	80.4
	Yes	44	30.8	83	13.5	19	6.50	64	19.6
	N/A	2	1.4	0	0	0	0	0	0
Resection status	R0	117	81.8	469	76.0	235	80.8	234	71.8
	R1	24	16.8	148	24.0	56	19.2	92	28.2
	N/A	2	1.4	0	0	0	0	0	0
Tumour regression status	TRG1	45	31.4					0	
	TRG2	34	23.8					154	47.2
	TRG3‡	64	44.8					172	52.8
Response	Responder	45	31.5					0	0
	(TRG1)								
	Non‐Responder	98	68.5					326	100
	(TRG2/3)‡								
EBV status	Positive	6§	4.2	24	3.9	8§	2.7	16	4.9
	Negative	137	95.8	593	96.1	283	97.3	310	95.1
MSI status	MSS	121	84.6	530	85.9	241	82.8	289	88.7
	MSI‐L	7§	4.9	28	4.5	15§	5.2	13	4.0
	MSI‐H	15	10.5	59	9.6	35	12.0	24	7.4

N/A, not available.

* OS was defined as time between the date of operation and death by any cause. For two patients who were not operated; the date of start of CTx was used.

† Classification according to 7th Edition UICC 2007.

‡ Two patients with tumour progression during CTx were not operated on; they were classified as TRG3 and a Non‐responder respectively.

§ One tumour biopsy and one resected tumour without neoadjuvant CTx were positive for both MSI‐L and EBV.

### Frequency of EBV and MSI in the biopsy and the resected tumour cohorts

EBV(+) was detected in 6 (4.2%) of the 143 tumour biopsies and MSI‐H and MSI‐L were found in 15 (10.5%) and 7 (4.9%) of the samples respectively (Table 1).

In the resected tumour cohorts, 24 (3.9%) of the 617 tumours were EBV positive, and 59 (9.6%) and 28 (4.5%) showed MSI‐H and MSI‐L, respectively (Table 1). Considering the type of unstable markers among the MSI‐L tumours, 33 (94%) of the 35 MSI‐L tumours showed instability at one of the three dinucleotide repeats that are included in the marker panel used for the determination of MSI.

The MSI status of the 42 paired biopsies before CTx and resected tumours after CTx were the same in all cases. None of these pairs was EBV(+).

All MSI‐H tumours were negative for EBV. One biopsy and one resected tumour were positive for both MSI‐L and EBV. These two patients were excluded from further analyses and clinical parameters were compared for the four molecular subgroups, EBV(+), MSI‐H, MSI‐L and MSS/EBV(−), taking the latter as reference.

### EBV, MSI and association with patient characteristics

Association with clinical characteristics was analysed for the 616 patients with resected tumours. EBV(+) was associated with male sex (*p* = 0.015), tumour localisation in the middle of the stomach (*p* = 0.033) and poor differentiation (*p* = 0.01). MSI‐H was associated with older age (*p* < 0.001), distal tumour localisation (*p* = 0.05) and absence of metastasis (*p* = 0.038). MSI‐L was more frequent in intestinal GC (*p* = 0.04) (see supplementary material, Table S3).

### EBV, MSI and response to neoadjuvant CTx

**Figure 2:**
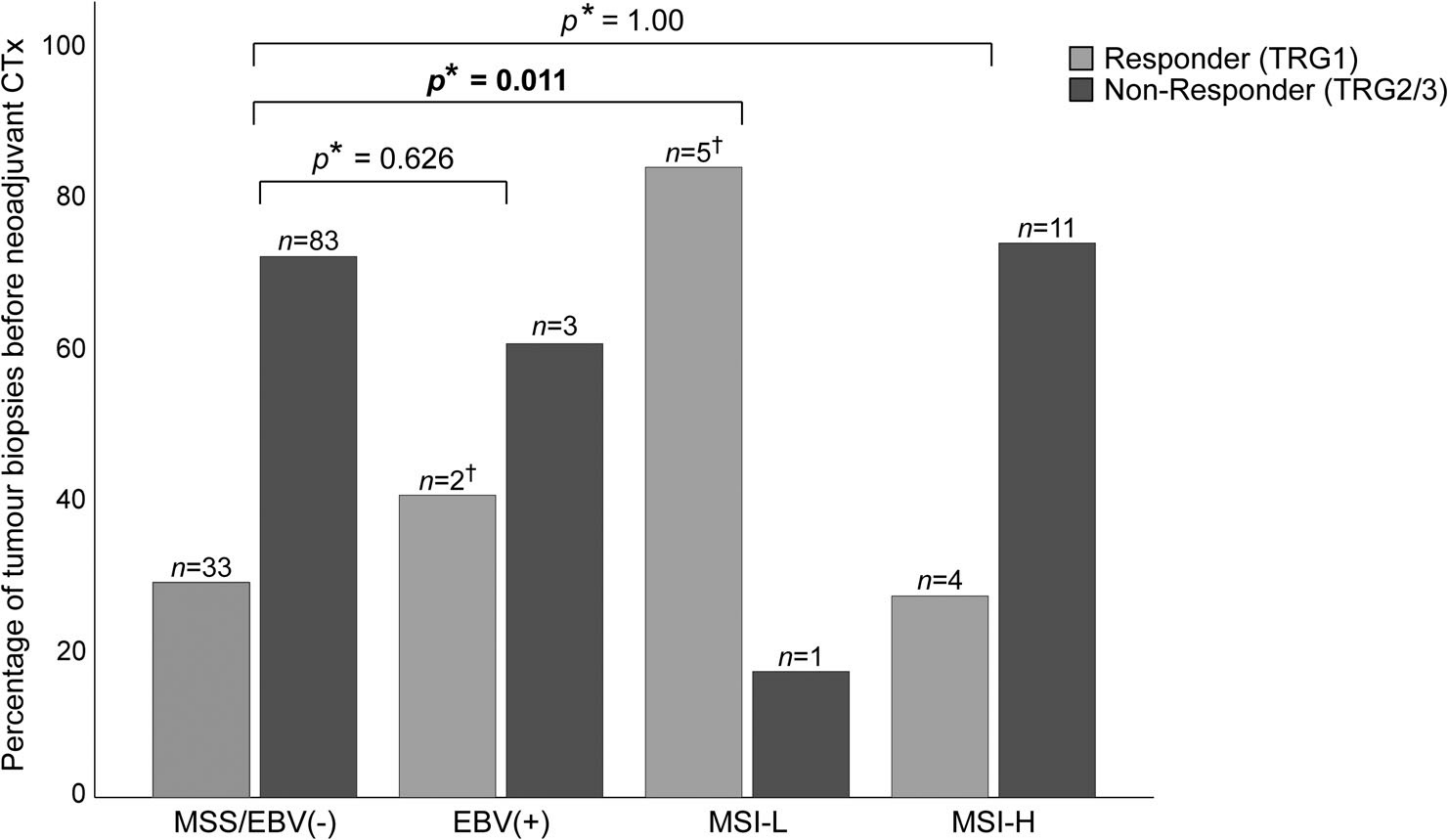
EBV and MSI status of pre‐therapeutic biopsies and response to neoadjuvant CTx. Comparison of the four molecular subgroups with response to neoadjuvant CTx is shown. *P value of Chi‐square test each compared with MSS/EBV(−). Significant value in bold. ^†^One tumour biopsy from a responding patient (TRG1) was positive for both MSI‐L and EBV, and was excluded from analysis.

EBV(+) and MSI‐H were not associated with response to CTx in the pre‐therapeutic biopsies before CTx (*p* = 0.626 and *p* = 1.00 respectively). In contrast, MSI‐L demonstrated a significant association with better response (*p* = 0.011). Five (83%) of six MSI‐L biopsies were of responding patients with TRG1 in the resected specimens after CTx compared to 33 (28%) of 116 MSS/EBV(−) tumours (Figure 2 and Table 2).

**Table 2:** EBV and MSI status of tumour biopsies before neoadjuvant CTx and resected tumours after neoadjuvant CTx and their association with response and tumour regression

	MSS/EBV(−) (n)	EBV(+) (n)	*P* value*	MSI‐L (n)	*P* value*	MSI‐H (n)	*P* value*
Tumour biopsies before neoadjuvant CTx (*n* = 142)							
Response							
Responder (TRG1)	33	2†	0.626	5†	**0.011**	4	1.00
Non‐responder (TRG2/3)	83	3		1		11	
Resected tumours after neoadjuvant CTx (*n* = 326)							
Tumour regression grade‡							
TRG2	136	8	0.989	6	0.796	4	**0.002**
TRG3	137	8		7		20	

* *P* value of Chi‐square test or Fisher's exact test compared to MSS/EBV(−).

† One tumour biopsy from a responding patient (TRG1) was positive for both; MSI‐L and EBV, and was excluded from analysis.

‡ TRG1 tumours were not included in the group of resected tumours after neoadjuvant CTx due to no or only extremely small amounts of residual tumour cells.

Significant *p* values are shown in bold.

Other groups reported differential responses of MSI‐H tumours to perioperative CTx 9 and we therefore additionally compared the prevalence of MSI‐H between the TRG2 and TRG3 group in our resected cohort after CTx. A significant difference was observed as in the TRG3 group 20 (12%) of 172 were MSI‐H compared to 4 (3%) of 154 in the TRG2 group (*p* = 0.002) (Table 2).

### EBV, MSI and survival in the biopsy cohort

**Figure 3:**
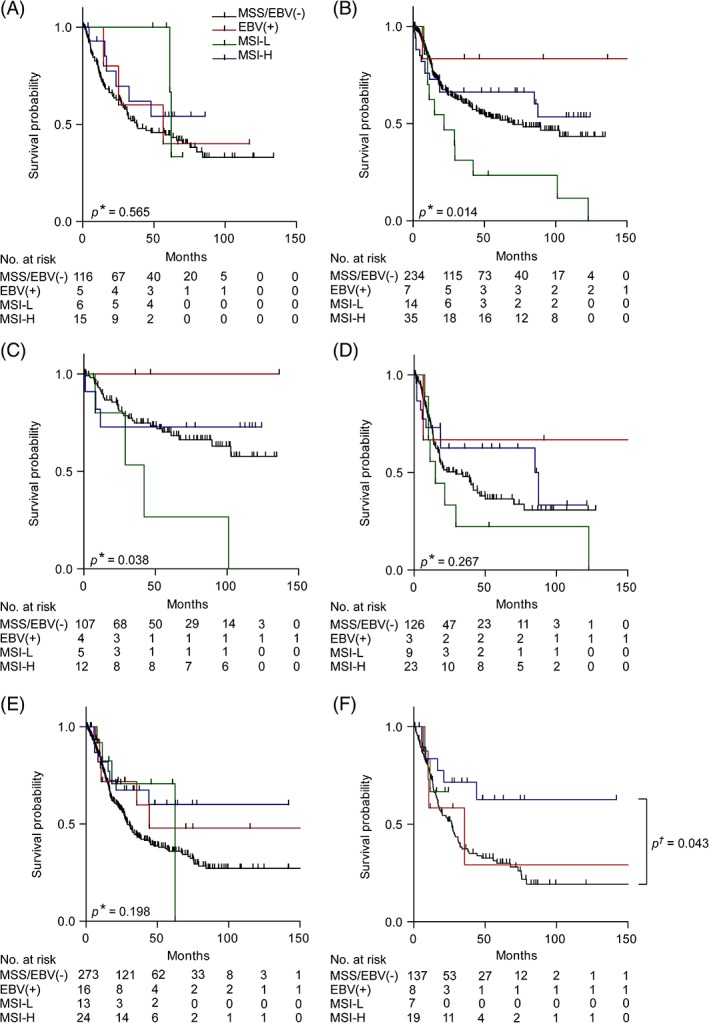
Discrimination of patient survival by EBV and MSI status. Kaplan–Meier curves of the EBV(+), MSI‐L, MSI‐H and MSS/EBV(−) patients are shown. Tumour biopsies before neoadjuvant CTx (A). Resected tumours from patients treated without neoadjuvant CTx: all patients (B), subgroups with clinical tumour stage cT2 (C) and cT3/cT4 (D). Resected tumours from patients after neoadjuvant CTx: all patients (E) and subgroup with TRG3 (F). *P value of log rank test (overall); ^†^ *P* value of Cox's regression.

Comparison of OS of patients with biopsies before CTx regarding the four subgroups showed no statistically significant difference (overall log rank *p* = 0.565) (Figure 3A). Essentially in line with an association of MSI‐L with better response to CTx, MSI‐L tumours showed the best OS (MSI‐L: HR, 0.47; 95% confidence interval [CI], 0.12–1.93, *p* = 0.297; MSI‐H: HR, 0.66, 95% CI, 0.29–1.53, *p* = 0.333; EBV(+): HR, 0.83, 95% CI, 0.26–2.64, *p* = 0.754). All survival data including the 1, 3 and 5 year OS rates are summarised in Table 3.

**Table 3:** Survival data of the patient cohorts and subgroups in association with EBV and MSI status

	EBV and MSI status	No.	Events	Survival probability (%)			Median survival (month)	HR	*P* value*
				1 year	3 years	5 years	(95% CI)	(95% CI)	
Tumour biopsies before neoadjuvant CTx	MSS/EBV(−)	116	66	74.6	52.0	44.6	37.9 (17.4–58.4)	1 ref.	
	EBV(+)	5	3	100	60.0	40	56.5 (0.0–123.7)	0.83 (0.26–2.64)	0.754
	MSI‐L	6	2	100	100	100	62.2 (60.6–63.9)	0.47 (0.12–1.93)	0.297
	MSI‐H	15	6	92.2	61.9	54.2	nr	0.66 (0.29–1.53)	0.333
	Total	142	77	78.3	55.1	47.3	48.1 (26.2–70.0)		
Resected tumours without neoadjuvant CTx (total)	MSS/EBV(−)	234	96	81.6	60.6	52.8	70.0 (32.7–107.3)	1 ref.	
	EBV(+)	7	1	83.3	83.3	83.3	nr	0.26 (0.04–1.87)	0.181
	MSI‐L	14	12	62.3	31.2	23.4	21.7 (1.0–42.4)	2.21 (1.21–4.04)	0.01
	MSI‐H	35	13	72.9	66.2	66.2	nr	0.81 (0.45–1.44)	0.465
	Total	290	122	79.7	60.4	53.8	85.0 (52.1–117.9)		
Resected tumours without neoadjuvant CTx (cT2)	MSS/EBV(−)	107	30	91.8	74.7	70.2	nr	1 ref.	
	EBV(+)	4	0	100	100	100	nr	0.43† (0–3.06)	0.495
	MSI‐L	5	4	80	53.3	26.7	42.2 (13.7–70.7)	3.88† (1.24–9.51)	0.023
	MSI‐H	12	3	72.7	72.7	72.7	nr	0.81† (0.22–2.20)	0.713
	Total	128	37	89.7	74.5	69.7	42.2(13.7–70.7)		
Resected tumours without neoadjuvant CTx (cT3/cT4)	MSS/EBV(−)	126	65	73.4	48.3	36.7	29.3 (11.4–47.2)	1 ref.	
	EBV(+)	3	1	66.7	66.7	66.7	nr	0.35 (0.05–2.56)	0.300
	MSI‐L	9	8	55.6	22.2	22.2	15 (4.2–25.8)	1.52 (0.72–3.20)	0.272
	MSI‐H	23	10	72.9	62.5	62.5	85 (20.1–149.9)	0.70 (0.36–1.36)	0.289
	Total	161	84	72.1	49.2	40.7	34.3 (17.6–51)		
Resected tumours after neoadjuvant CTx (total)	MSS/EBV(−)	273	152	77.4	43.3	36	29.1 (24.6–33.6)	1 ref.	
	EBV(+)	16	6	71.8	59.8	47.9	44.4	0.64 (0.29–1.46)	0.290
	MSI‐L	13	4	82.5	70.7	70.7	62.4	0.64 (0.24–1.74)	0.385
	MSI‐H	24	8	81.9	67.5	60	nr	0.54 (0.26–1.09)	0.085
	Total	326	170	77.7	46.5	39	32.4 (23.0–41.8)		
Resected tumours after neoadjuvant CTx (TRG3)	MSS/EBV(−)	137	83	73.3	37.3	30	26.7 (19.2–34.2)	1 ref.	
	EBV(+)	8	4	58.3	29.2	29.2	35.6 (0–73.6)	0.91 (0.33–2.47)	0.847
	MSI‐L	7	2	66.7	0	0	nr	0.79 (0.19–3.21)	0.736
	MSI‐H	19	6	82.5	69.8	59.9	nr	0.43 (0.19–0.98)	0.043
	Total	171	95	73.2	41.0	33.4	27.4 (20.8–34.0)		

ref., reference; nr, not reached.

* *P* value of Cox's regression.

† HRs were calculated according to Firth's correction.

### EBV, MSI and survival in the resected non‐CTx cohort

Analysis of OS of patients in the resected cohort was separately performed in the groups stratified according to CTx (yes/no). In the non‐CTx group a statistically significant difference of OS regarding the four molecular groups was observed (overall log rank *p* = 0.014) (Figure 3B). Patients with EBV(+) tumours showed the best OS followed by MSI‐H tumours (EBV(+): HR, 0.26; 95% CI, 0.04–1.87, *p* = 0.181; MSI‐H: HR, 0.81; 95% CI, 0.45–1.44, *p* = 0.465). Patients with MSI‐L tumours showed a statistically significantly worse OS (HR, 2.21; 95% CI, 1.21–4.04, *p* = 0.01) (Table 3).

Subgroup analysis within the resected non‐CTx cohort stratified according to clinical tumour stage demonstrated a pronounced difference – especially regarding MSI‐L – in the cT2 group (overall log rank *p* = 0.038) (Figure 3C,D). All patients with EBV(+) tumours were alive and patients with MSI‐L tumours showed a significantly worse OS compared to the MSS/EBV(−) negative tumours (EBV(+): HR, 0.43; 95% CI, 0–3.06, *p* = 0.495; MSI‐H: HR, 0.81, 95% CI, 0.22–2.20, *p* = 0.713; MSI‐L: HR, 3.88; 95% CI, 1.24–9.51, *p* = 0.023) (Table 3).

Multivariable analysis was performed for the total resected non‐CTx cohort. Analysing the molecular subgroups and the pre‐therapeutically available clinical factors (age, sex, cT, histological type according to Laurén, tumour localisation) revealed cT (*p* < 0.001), age (*p* = 0.001) and the molecular classification (overall *p* = 0.027) as significant prognostic factors. Interestingly, considering the molecular subgroups separately, MSI‐H emerged as an independent prognostic factor (Table 4).

**Table 4:** Multivariable analysis of survival including pre‐ and post‐therapeutically available clinical factors in the resected non‐CTx cohort

	HR	95% CI	*P* value*
Pre‐therapeutic factors†			
Clinical tumour stage			
cT2	1	–	<0.001
cT3/4	2.74	1.84–4.07	
Age	1.03	1.01–1.05	0.001
Molecular classification			0.027
MSS/EBV(−)	1	–	–
EBV(+)	0.20	0.03–1.46	0.113
MSI‐L	1.53	0.83–2.84	0.175
MSI‐H	0.55	0.30–1.00	0.049
Post‐therapeutic factors‡			
pN			
pN0	1	–	<0.001
pN1	3.15	1.95–5.10	
Age	1.03	1.01–1.04	0.004
Resection status			
R0	1	–	0.020
R1	1.68	1.08–2.60	
Localisation			0.026
Proximal	1	–	–
Middle	0.67	0.42–1.05	0.079
Distal	0.52	0.33–0.83	0.006
Total	1.09	0.51–2.31	0.830
Post‐therapeutic factors (R0 resected, non‐CTx cohort)			
pN			
pN0	1	–	<0.001
pN1	2.67	1.59–4.48	
Age	1.03	1.01–1.05	0.004
Molecular classification			0.035
MSS/EBV(−)	1	–	–
EBV(+)	0.23	0.03–1.66	0.144
MSI‐L	1.80	0.88–3.71	0.110
MSI‐H	0.55	0.28–1.10	0.090
pT§			
pT1/2	1	–	0.023
pT3/4	1.36	1.04–1.78	

* *P* value of forward likelihood ratio Cox's regression model.

† Pre‐therapeutic factors included: age, sex, localisation, Laurén subtypes, clinical tumour stage, molecular classification.

‡ Post‐therapeutic factors included: age, sex, localisation, Laurén subtypes, pT, pN, M‐status, R‐status, molecular classification.

§ Classification according to 7th Edition UICC 2007.

Including the post‐therapeutically available factors revealed only the clinical parameters pN (*p* < 0.001), age (*p* = 0.004) and R‐category (*p* = 0.020) as independent prognostic factors (Table 4). Analysis of the subgroup of only completely resected patients (R0 group) revealed pN (*p* < 0.001), age (*p* = 0.004), the molecular classification (*p* = 0.035) and pT (*p* = 0.023) as independent prognostic factors (Table 4).

### EBV, MSI and survival in the resected cohort after neoadjuvant CTx

In the CTx group, differences in OS were not statistically significant (overall log rank *p* = 0.198) (Figure 3E). However, an obviously better OS was observed for patients with MSI‐H tumours (HR, 0.54; 95% CI, 0.26–1.09, *p* = 0.085). These results are included in Table 3.

Subgroup analysis in the TRG2 and TRG3 groups separately revealed a significantly better OS for patients with MSI‐H tumours in the TRG3 group (HR, 0.43; 95% CI, 0.19–0.98, *p* = 0.043) (Figure 3F and Table 3).

## Discussion

Molecular subtypes in GC have been identified, but knowledge about their clinical relevance in particular in the context of preoperative CTx is limited 4, 5, 9, 14, 17, 25, 26, 27. In this study, we addressed this issue and analysed the prognostic and predictive significance of four molecular subgroups namely EBV(+), MSI‐H, MSI‐L and MSS/EBV(−) in pre‐therapeutic tumour biopsies of GC patients before platinum/5‐FU based neoadjuvant CTx and in resected tumours of patients with or without neoadjuvant CTx.

One of the most interesting finding of our study was a better response to neoadjuvant CTx of MSI‐L tumours in the pre‐therapeutic biopsy cohort. The patients also showed an increased OS, although the difference was statistically not significant, likely due to low‐sample size. Interestingly, MSI‐L seems to have a differential prognostic role depending on the specific treatment of the patients as in our resected cohort treated with surgery alone MSI‐L demonstrated a negative prognostic effect. Usually, pre‐/perioperative CTx is recommended for patients with advanced tumour stages (cT3/cT4), but some experts endorse that patients with cT2 tumours can also be treated 2, 28. As clinical staging is relatively imprecise and the negative prognostic effect of MSI‐L was particularly prominent for patients with clinically staged cT2 tumours in our study, the determination of MSI‐L may contribute to improved management of GC patients in this context. The analysis of MSI‐L is based on a simple, cost efficient multiplex PCR assay. Thus, assuming confirmation by other studies, MSI‐L could represent an attractive marker for routine clinical application, even considering the relatively low number of 4–5% of patients demonstrating the MSI‐L phenotype in their tumours. MSI‐L has been detected in various tumour entities including GC over a range of 4–20% 7, 8, 29. However, it has to be emphasised that the detection rate of MSI‐L is dependent on the number and on the type of the microsatellite markers tested 30. In our study, instability in MSI‐L tumours was mainly restricted to alterations at dinucleotide repeat markers. An association of MSI‐L with MSI preferentially at dinucleotide repeats and with worse prognosis has been demonstrated in colorectal cancer, which is compatible with our results 29.

In contrast to the MSI‐H phenotype with a well‐known molecular background related to defects in one of the four DNA mismatch repair genes MLH1, MSH2, MSH6 and PMS2, the origin and biological significance of the MSI‐L phenotype is largely unclear and controversially discussed. MSI‐L has been related to elevated mutation rates, to defects in specific DNA repair genes and/or induction by DNA damaging agents 30, 31. Based on our results, it is tempting to speculate that MSI‐L may reflect a particular type of impaired DNA repair and numerous proteins involved in these complex mechanisms represent possible candidates in that scenario. Comparing the frequencies of MSI‐L among the pre‐therapeutic biopsies and the resected tumours after CTx in our study, one could expect a decrease of MSI‐L in resected tumours after CTx given the association of MSI‐L with tumours of responding patients, which are not present in the resected tumour group. However, we found only a slight difference (4.90% in the biopsies, 3.98% in the resected tumours). Although highly speculative, induction of MSI‐L by a DNA damaging agent or tumour heterogeneity may counteract this assumed decrease.

Regarding MSI‐H, the majority of studies has demonstrated an association of this type of MSI with good prognosis in GC, which is essentially in line with our findings for patients in the resected cohorts 13, 27, 32, 33. In the context of CTx, however, different results have been reported 8, 9, 11, 12, 14. An attenuated or negative prognostic effect of MSI‐H has recently been reported for patients treated with adjuvant CTx 11, 34, 35. In addition, a negative prognostic effect of MSI based on the analysis of the resected tumours after CTx was proposed for patients who underwent preoperative CTx in the MAGIC trial 9. Our findings of no negative prognostic significance of MSI‐H when pre‐therapeutic tumour biopsies before CTx were analysed and the good prognostic effect of MSI‐H in the groups of resected patients both with and without CTx, do not support these results. Our data from tumour biopsies before neoadjuvant CTx may allow for a more comprehensive conclusion about the relevance of MSI‐H for response in terms of tumour regression and OS in the setting of neoadjuvant CTx than an analysis of resected specimens after CTx, in which tumours from patients with near to complete and complete response are naturally missing. Comparing the frequency of MSI‐H between the tumours with TRG2 and TRG3 after CTx in our study, we found an enrichment of MSI‐H in the TRG3 group. This is somewhat in line with the data from others mentioned above 9 and, indeed, argues for an association of MSI‐H with compromised response. However, patients with MSI‐H tumours in the TRG3 group still showed a significantly better OS than MSS/EBV(−) patients, thus underlining the positive prognostic effect of MSI‐H, even for patients with no or only minor response to CTx. This is essentially in line with a recent study analysing gastric and gastro‐oesophageal junction cancer patients undergoing neoadjuvant CTx 14. The frequency and the significant associations of MSI‐H which we found with patient age, tumour location or status of metastasis confirms results reported by others 13, 34.

EBV was detected in 4–5% of our tumours, which is similar to a recent report 10, 16. We did not observe an association of EBV(+) with response to CTx. However, in the non‐CTx resected cohort a better OS was observed for patients with EBV(+) tumours. In addition, an association of EBV(+) with tumour location and male sex was found, which is in line with results reported by others 10, 25.

Regarding multivariable analysis for survival performed in the non‐CTx cohort, our results confirm the well‐known prognostic impact of lymph node involvement and completeness of tumour resection as independent prognostic factors 36. The prognostic relevance of our molecular classification was underlined as it emerged as an independent prognostic factor in the multivariable analysis of only the completely resected patients and when considering only pre‐therapeutically available factors.

Despite the comprehensive analysis of a very large cohort of patients comprising 760 tumour samples overall, our study has limitations which are mainly related to its retrospective nature. Regarding the analysis of the pre‐therapeutic biopsies, the availability of DNA or suitable tumour tissues presented the main limiting factor for inclusion of patients. Further limitations are that our analysis was not performed in the context of a randomised clinical trial testing different treatment regimens but refers to a sample series from daily clinical practice of a local hospital with some variations regarding surgical approaches and treatment protocols. Thus, our study has to be considered an explorative analysis. Further prospective studies are needed to confirm our results and a comprehensive molecular analysis of MSI‐L tumours should be performed to clarify the biological background of this particular type of MSI.

To conclude, in our study MSI‐H and EBV were not predictive of response to neoadjuvant platinum/5‐FU based CTx, but they were indicative of a good prognosis. In particular, considering MSI‐H, this was evident in principal regardless of the therapeutic approach chosen. MSI‐L, however, was predictive of good response to CTx. Furthermore, the negative prognostic effect of MSI‐L observed for patients treated with surgery alone, even in the group with clinically determined earlier tumour stages, indicates that MSI‐L might help to delineate patients with potentially high‐benefit from preoperative platinum/5‐FU based treatment. Clearly, additional studies are mandatory to confirm our results and the MSI‐L phenotype warrants further investigation to elucidate its role in chemosensitivity and tumour development.

## Author contributions statement

MKo, BG, WW and GK planned and conducted the study. MKo, BG, MKr, SB, AN, MR, TS, LI, DM, PM, MMG and LB collected the data. MKo, BG, MKr, JSH, MJ, AN, AH, KO, WW, GK interpreted the data. MKo, BG and GK drafted the manuscript. MJ, AN, AH, MMG, KO and WW reviewed the manuscript. All authors have approved the final drafts submitted.

## Supporting information


**Supplementary Material and Methods**
**Figure S1.** Discrimination of patient survival by tumour regression grade (TRG)
**Table S1.** Chemotherapy regimens of the preoperatively treated patients
**Table S2.** Drug regimens and survival of the preoperatively treated patients
**Table S3.** EBV and MSI status of resected tumours without and after neoadjuvant CTx and association with patient`s characteristics
