# Supplementary material for: Prognostic implication of molecular subtypes and response to neoadjuvant chemotherapy in 760 gastric carcinomas: role of Epstein–Barr virus infection and high‐ and low‐microsatellite instability
Source: J Pathol Clin Res. 2019 Jun 17;5(4):227–39. doi: 10.1002/cjp2.137 (PMC6817827; doi:10.1002/cjp2.137)
Supplement: Supplementary file 1 — Supplementary Material and Methods Figure S1. Discrimination of patient survival by tumour regression grade (TRG) Table S1. Chemotherapy regimens of the preoperatively treated patients Table S2. Drug regimens and survival of the preoperatively treated patients Table S3. EBV and MSI status of resected tumours without and after neoadjuvant CTx and association with patient`s characteristics [file CJP2-5-227-s001.docx]

**Prognostic implication of molecular subtypes and response to neoadjuvant chemotherapy in 760 gastric carcinomas: role of Epstein Barr virus infection and high and low microsatellite instability**

Kohlruss M *et al*. *J Path: Clin Res* DOI: 10.1002/cjp2.137

**Supplementary Material**

**Prognostic implication of molecular subtypes and response to neoadjuvant chemotherapy in 760 gastric carcinomas: role of Epstein Barr virus infection and high and low microsatellite instability**

Meike Kohlruss, Bianca Grosser, Marie Krenauer, Julia Slotta-Huspenina, Moritz Jesinghaus, Susanne Blank, Alexander Novotny, Magdalena Reiche, Thomas Schmidt, Liridona Ismani, Alexander Hapfelmeier, Daniel Mathias, Petra Meyer, Matthias M. Gaida, Lukas Bauer, Katja Ott, Wilko Weichert and Gisela Keller

**Supplementary Materials and Methods (reference numbers refer to the main text)**

**Surgery type**

For gastric cancer, total or subtotal distal gastrectomy with D2 lymphadenectomy according to Japanese guidelines was undertaken depending on the location of the tumour [22]. The tail of the pancreas and the spleen were only resected when directly involved by the tumour. For Siewert type II and III cancers of the gastro-oesophageal junction (GEJ), gastrectomy with transhiatal distal abdominal oesophagectomy plus D2 lymphadenectomy was performed. If the oral margin of the tumour could not be safely reached via a transhiatal abdominal approach in GEJ type II tumours, a right thoracoabdominal oesophagectomy with resection of the lesser curvature and fundus of the stomach with 2-field (mediastinal and abdominal) lymphadenectomy (Ivor-Lewis procedure) including an intraabdominal D2 lymphadenectomy was performed [37]. Comparison of OS of patients treated with gastrectomy versus those treated with oesophagectomy or other surgical approaches revealed no statistically significant differences (*p* = 0.75).

**DNA isolation**

Tissue samples with only small amounts were re-suspended after manual microdissection in 200 µl of 50 mmol/L Tris-HCl pH 8.5, 1 mmol/L of EDTA, 0.5% of Tween 20, 0.2 mg/ml of proteinase K and incubated at 55°C for 3 hours. Proteinase K was inactivated by boiling for 10 minutes and a 1:3 – 1:10 dilution was directly used for PCR. Normal and tumour tissue DNA concentrations were determined by measuring the absorbance at 260 nm using a NanoDrop spectrophotometer (Thermo Scientific, Waltham, MA) or the Qubit DNA quantitation assay (Thermo Scientific, Waltham, MA).

## Analysis for microsatellite instability (MSI)

MSI was analysed using the five markers BAT25, BAT26, D2S123, D5S346 and D17S250 recommended by the National Cancer Institute [6]. A multiplex PCR with fluorescence-tagged primers was performed using the Type-it Microsatellite PCR kit (Qiagen, Hilden, Germany) on non-tumorous and tumour DNA. 20 ng DNA was added to each PCR reaction in a final volume of 25µl. Cycle conditions were as follows: after an initial step of 95°C for 5 min, 32 cycles were performed consisting of denaturation at 95°C for 30 sec, annealing at 58°C for 90 sec and extension at 72°C for 30 sec and final extension at 60°C for 30 min. Separation and detection of the PCR products were performed in a 3130 Genetic Analyzer (Applied Biosystems, Foster City, CA) loaded with POP-7 polymer (Applied Biosystem) and using ROX-500 Genescan (Thermo Scientific) as size standard. Samples were analysed with the GeneMapper Software 5 (Applied Biosystem).

**Supplementary Figure and Tables**


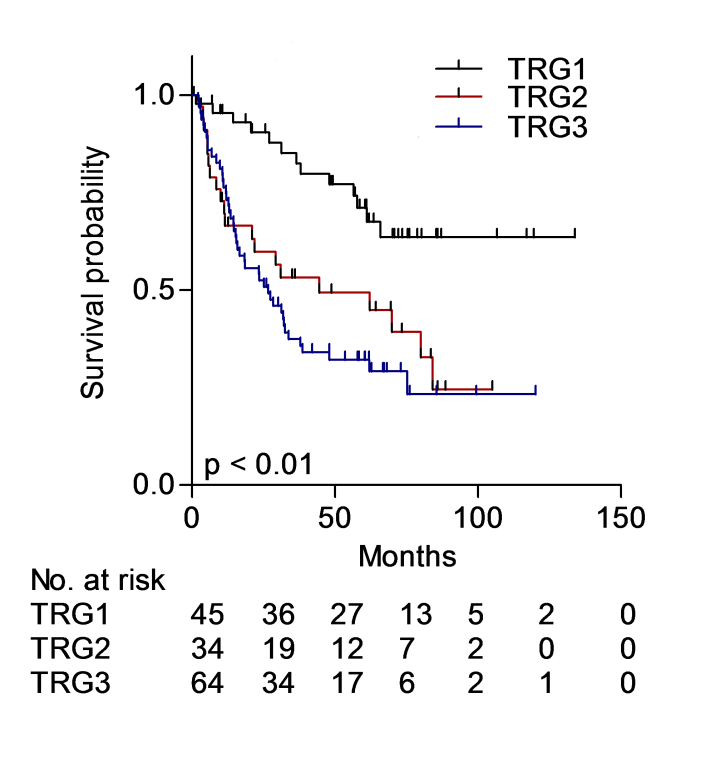


**Figure S1:** Discrimination of patient survival by tumour regression grade (TRG).

Kaplan-Meier curves of tumour biopsies before neoadjuvant CTx with TRG1, TRG2 and TRG3 are shown.

**Table S1.** Chemotherapy regimens of the preoperatively treated patients

|  |  | **Tumour biopsies before CTX** | | **Resected tumours after CTx** | |
| --- | --- | --- | --- | --- | --- |
|  |  | ***n*** | **%** | ***n*** | **%** |
| **Total** |  | 143 | 100 | 326 | 100 |
| **Preoperative chemotherapy** | Cis + 5-FU or Cap | 117 | 82 | 124 | 38 |
|  | Ox + 5-FU or Cap | 18 | 13 | 46 | 14 |
|  | Cis + 5-FU + Doc or Pac | 2 | 1 | 27 | 8 |
|  | Ox + 5-FU + Doc | 0 | 0 | 21 | 6 |
|  | Cis or Ox + 5-FU or Cap + Epi | 5 | 4 | 84 | 26 |
|  | Others | 1 | <1 | 23 | 7 |
|  | n/a | 0 | 0 | 1 | <1 |

Cis, cisplatin; Ox, oxaliplatin; 5-FU, 5-fluorouracil; Cap, capecitabine; Doc, docetaxel; Pac, paclitaxel; Epi, epirubicin; Others, combination of Cis/Ox with other agents or cross over between different treatment regimens;

n/a, no data available

**Table S2.** Drug regimens and survival of the preoperatively treated patients

| **Subgroup** | **Factor** | **Cases**  **(No.)** | **Median**  **Survival [mo]**  **(95% CI)** | ***P* value^1^** |
| --- | --- | --- | --- | --- |
|  | **CTx regimens: Platin based or**  **Platin and Taxane^2^** | | |  |
| Resected specimens after neoadjuvant CTx  (TRG2/3) | Platin based | 254 | 29.1 (22.4 – 35.8) | 0.43 |
|  | Platin + Taxane | 48 | 44.4 (9.1 – 79.7) |  |
|  | Others | 24 | 32.4 (4.7 – 60.0) |  |
| Tumour biopsies  before neoadjuvant CTx  (TRG1 and TRG2/3) | Platin based | 140 | not reached | 0.27 |
|  | Platin + Taxane | 2 | not reached |  |
|  | Others | 1 | not reached |  |
|  | **CTx regimens with two or**  **three agents^3^** | | | |
| Resected specimens after neoadjuvant CTx (TRG2/3) | Two agents | 171 | 26.7 (19.5 – 33.9) | 0.27 |
|  | Three agents | 133 | 44.1 (26.7 – 61.5) |  |
|  | Others | 22 | 32.4 (23.0 – 41.8) |  |
| Tumour biopsies  before neoadjuvant CTx  (TRG1 and TRG2/3) | Two agents | 135 | not reached | 0.56 |
|  | Three agents | 7 | not reached |  |
|  | Others | 1 | not reached |  |

^1^*P* value of log rank test (overall)

^2^According to supplementary Table S1: Platin based (PLF, CAPOX, OLF, FUFOX, FOLFOX, E-PLF/EOX/ECX,); Platin + Taxan (Taxol-PLF, Taxotere-PLF, FLOT, DCF, TCF); others: Platin + Imatinib/Panitumomab/Cetuximab

^3^According to supplementary Table S1: Two agents (PLF, CAPOX, OLF, FUFOX, FOLFOX); three agents (Taxol-PLF, Taxotere-PLF, FLOT, E-PLF/EOX/ECX, DCF, TCF); others: Platin + Imatinib/Panitumomab/Cetuximab

**Table S3.** EBV and MSI status of resected tumours without and after neoadjuvant CTx, and association with patient characteristics

|  |  | Resected tumours (*n*=616)^1^ | | | | | | |
| --- | --- | --- | --- | --- | --- | --- | --- | --- |
| Category | **Value** | **MSS/EBV(-)**  ***n*** | **EBV(+)**  ***n*** | ***P* value**^2^ | **MSI-L**  ***n*** | ***P* value**^2^ | **MSI-H**  ***n*** | ***P* value**^2^ |
| Cases | Total | 507 | 23 |  | 27 |  | 59 |  |
| Age | Median | 63.9 | 57.6 |  | 66.5 |  | 71.4 |  |
|  | Range | 28.3-90.9 | 29.2-80.9 |  | 49.3-82.2 |  | 40.4-84.9 |  |
| Age Median | < Median | 266 | 16 | 0.108 | 11 | 0.235 | 13 | <0.001 |
|  | ≥ Median | 241 | 7 |  | 16 |  | 46 |  |
| Sex | Male | 370 | 22 | 0.015 | 23 | 0.161 | 38 | 0.165 |
|  | Female | 137 | 1 |  | 4 |  | 21 |  |
| Localization | Proximal | 255 | 9 | 0.033 | 14 | 1.00 | 23 | 0.05 |
|  | Middle/Total | 120 | 12 |  | 7 |  | 14 |  |
|  | Distal | 102 | 2 |  | 5 |  | 21 |  |
|  | Total/linitis | 26 | 0 |  | 1 |  | 1 |  |
|  | n/a | 4 | 0 |  | 0 |  | 0 |  |
| Laurén histological  subtype | Intestinal | 273 | 14 | 0.509 | 20 | 0.04 | 39 | 0.073 |
|  | Non intestinal | 234 | 9 |  | 7 |  | 20 |  |
| Tumour grade | G1/2 | 109 | 0 | 0.01 | 4 | 0.347 | 11 | 0.676 |
|  | G3/4 | 324 | 18 |  | 20 |  | 38 |  |
|  | n/a | 74 | 5 |  | 3 |  | 10 |  |
| cT | cT2 | 121 | 5 | 0.807 | 5 | 0.517 | 12 | 0.535 |
|  | cT3/4 | 384 | 18 |  | 22 |  | 47 |  |
|  | n/a | 2 | 0 |  | 0 |  | 0 |  |
| (y) pT^3^ | (y) pT1/2 | 112 | 6 | 0.652 | 6 | 0.987 | 11 | 0.543 |
|  | (y) pT3/4 | 395 | 17 |  | 21 |  | 48 |  |
| (y) pN | Negative | 151 | 9 | 0.340 | 8 | 0.986 | 21 | 0.358 |
|  | Positive | 356 | 14 |  | 19 |  | 38 |  |
| Metastasis status | No | 431 | 22 | 0.228 | 24 | 0.783 | 56 | .038 |
|  | Yes | 76 | 1 |  | 3 |  | 3 |  |
| Neoadjuvant Chemotherapy | No | 234 | 7 | 0.139 | 14 | 0.563 | 35 | .055 |
|  | Yes | 273 | 16 |  | 13 |  | 24 |  |

EBV(-), Epstein-Barr virus negative; EBV(+), Epstein-Barr virus positive; MSS, microsatellite stable; MSI-L, low microsatellite instability; MSI-H, high microsatellite instability; n/a, not available

^1^One resected tumour was positive for both, MSI-L and EBV and was excluded from analysis.

^2^*P* value of Chi-Square test or Fisher’s exact test compared to MSS/EBV(-).

^3^Classification according to 7^th^ Edition UICC 2007.
